# Crystal structure of (*E*)-2-[(2-hy­droxy-4-meth­oxy­phen­yl)(phen­yl)methyl­idene]-*N*-phenyl­hydrazine-1-carboxamide

**DOI:** 10.1107/S2056989015005757

**Published:** 2015-03-28

**Authors:** C. F. Annie, M. Sithambaresan, M. R. Prathapachandra Kurup

**Affiliations:** a Department of Applied Chemistry, Cochin University of Science and Technology, Kochi 682 022, India; b Department of Chemistry, Faculty of Science, Eastern University, Chenkalady, Sri Lanka

**Keywords:** crystal structure, hydrazinecarboxamide, supra­molecular, hydrogen bonding, C=O⋯π inter­actions, π–π inter­actions

## Abstract

The title compound, C_21_H_19_N_3_O_3_, has an *E* conformation about the azomethine double bond. The central moiety of the hydrazinecarboxamide moiety [–N—N—C(=O)—N–] has an almost coplanar arrangement [maximum deviation for the C atom = 0.010 (2) Å]. This central moiety is flanked by three aromatic rings and its mean plane makes dihedral angles of 24.7 (1), 72.91 (12) and 34.26 (11) Å, respectively, with the phenolic ring, the phenyl ring attached to the same C atom as the phenolic ring, and the phenyl­hydrazine ring. The adjacent phenolic and phenyl rings are twisted away from each other to reduce steric hindrance and make a dihedral angle of 80.59 (12)°. The phenolic and phenyl­hydrazine rings are inclined to one another by 28.89 (11)°. The rigidity of the mol­ecule is increased by an intra­molecular O—H⋯N hydrogen bond involving the phenolic hydrogen and the azomethine N atom. In the crystal, the carbonyl O atom forms bifurcated hydrogen bonds with the two NH atoms of the hydrazinic group, leading to the formation of chains propagating along [001]. Within the chains there are also C—H⋯O hydrogen bonds present. The chains are linked *via* C=O⋯π [3.4316 (18) Å] and parallel slipped π–π inter­actions, involving inversion-related benzene rings [centroid–centroid distance = 3.8850 (14) Å; inter-planar distance = 3.3895 (10) Å; slippage = 1.899 Å], forming sheets lying parallel to (100).

## Abstract 2

The title compound, has an *E* conformation about the azomethine double bond and an intra­molecular O—H⋯N hydrogen bond involving the phenolic hydrogen and the azomethine N atom. In the crystal, mol­ecules are linked by bifurcated N—H⋯O hydrogen bonds involving the same acceptor atom, forming chains propagating along [001].

## Chemical context   

Semicarbazones are urea derivatives exhibiting a wide spectrum of biological activities (Beraldo & Gambino, 2004 ▸). They have been found to be associated with anti­tumoral (Afrasiabi *et al.*, 2005 ▸), anti­microbial (Siji *et al.*, 2010 ▸), anti­hypertensive, hypolipidemic, anti­neoplastic, hypnotic and anti­convulsant properties. They can function as excellent ligands to various metal ions (Kala *et al.*, 2007 ▸; Aiswarya *et al.*, 2013 ▸; Kurup *et al.*, 2011 ▸) and can coordinate to metal ions either in the neutral (Siji *et al.*, 2011 ▸) or in the anionic forms (Reena *et al.*, 2008 ▸). Single crystals of aceto­phenone semicarbazones are potential organic non-linear optical (NLO) materials and they have a wide transparency window in the entire visible region, making them ideal candidates for NLO device applications (Vijayan *et al.*, 2001 ▸). Semicarbazones have been proposed as analytical reagents that can be used in selective and sensitive determination of metal ions (Garg & Jain, 1988 ▸). The crystal structure of the dimethylformamide solvate of the title compound has been reported (Annie *et al.*, 2012 ▸).
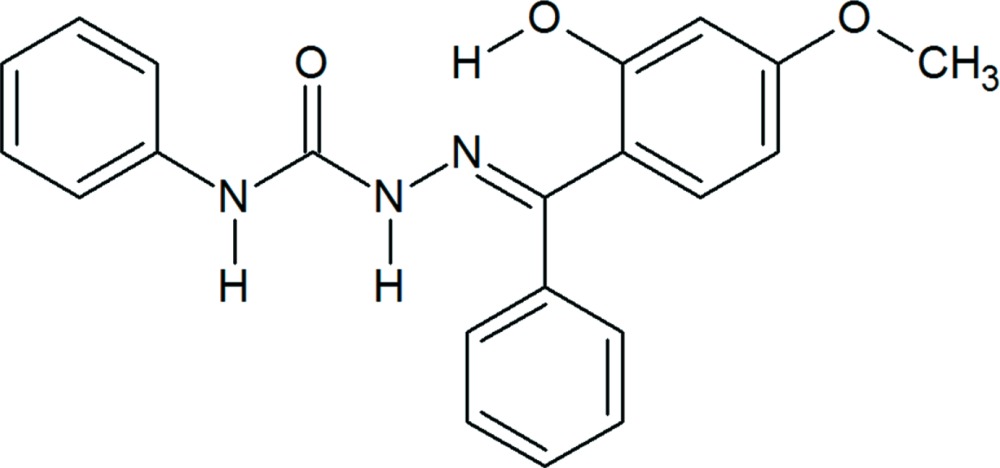


## Structural commentary   

**Figure 1:**
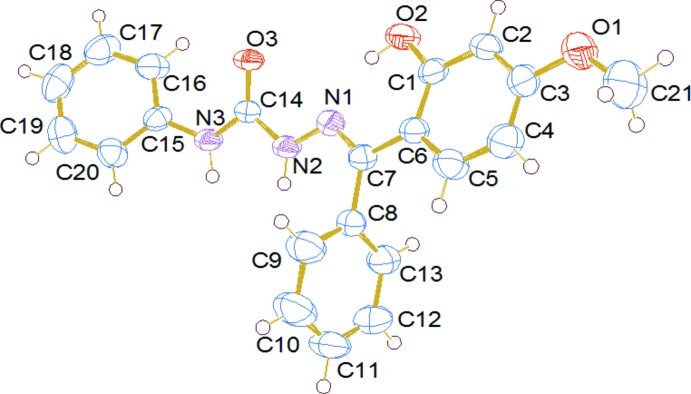
A view of the mol­ecular structure of the title compound, with the atom labelling. Displacement ellipsoids are drawn at the 50% probability level.

**Figure 2:**
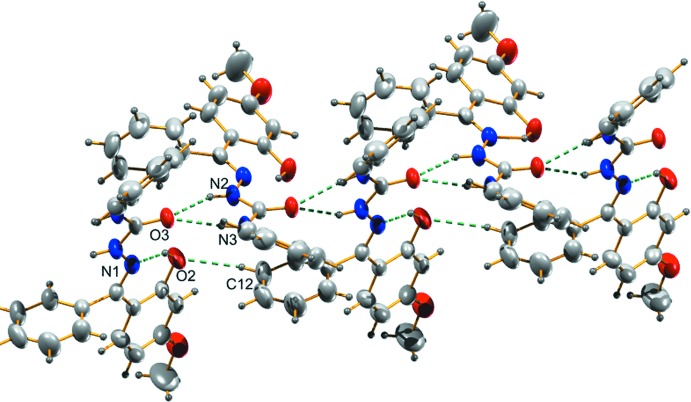
A view of the hydrogen-bonding inter­actions (dashed lines) in the title compound, forming chains propagating along [001] (see Table 1 ▸ for details).

In the mol­ecule of the title compound (Fig. 1 ▸), the conform­ation about the C7=N1 bond is *E*, and the central hydrazinecarboxamide moiety [–N1—N2—C14(=O3)—N3–] is almost planar [the maximum deviation is 0.010 (2) Å for atom C14]. This central moiety is flanked by three aromatic rings (C1–C6, C8–C13 and C15–C20) which are inclined to its mean plane by 24.70 (10), 72.91 (12) and 34.26 (11) °, respectively. Rings C1–C6 and C8–C13, attached at the same C atom (C7), are twisted away from each other and make a dihedral angle of 80.59 (12)°. They are inclined to the phenyl­hydrazine ring (C15–C20) by 28.89 (11) and 52.42 (12)°, respectively. In the crystal structure of the dimethylformamide solvate of the title compound (Annie *et al.*, 2012 ▸), the two rings attached at the same C atom (C7) are inclined to one another by 88.47 (10)°, while they are inclined to the phenyl­hydrazine ring by 14.42 (10)° for the phenolic ring, and by 82.35 (11)° for the phenyl ring. There is an intra­molecular O—H⋯N hydrogen bond (Fig. 2 ▸) involving the phenolic hydrogen and the azomethine atom N1 (Fig. 2 ▸ and Table 1 ▸). This hydrogen bond is also present in the structure of the dimethylformamide solvate of the title compound mentioned above.

## Supra­molecular features   

**Figure 3:**
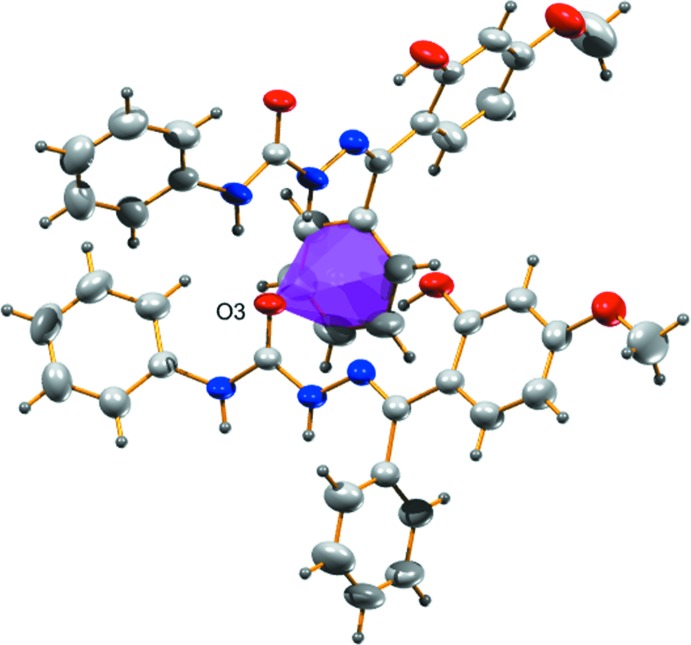
C=O⋯π inter­action in the crystal structure of the title compound.

**Figure 4:**
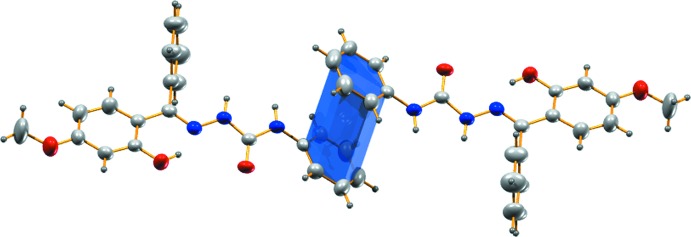
π–π inter­action in the crystal structure of the title compound.

**Figure 5:**
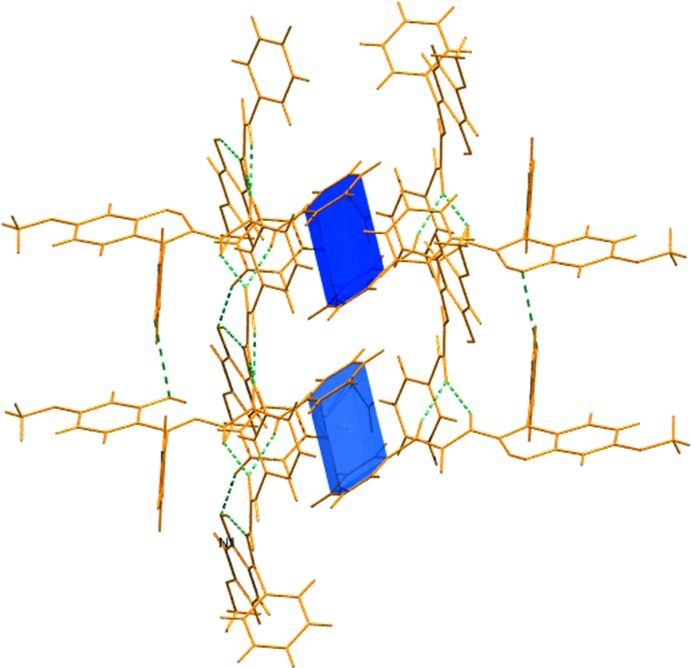
A view along the *a* axis of the formation of the sheets lying parallel to (100) in the crystal structure of the title compound.

In the crystal, the carbonyl O atom (O3) acts as the acceptor in bifurcated hydrogen bonds with the NH atoms of atoms N2 and N3 of the hydrazinic group, leading to the formation of chains propagating along [001]; Table 1 ▸ and Fig. 2 ▸. Within the chains there are also C—H⋯O hydrogen bonds present (Table 1 ▸ and Fig. 2 ▸). The chains are linked *via* C14=O3⋯π inter­actions [distance O3⋯*Cg* ^i^ = 3.4316 (18) Å; angle C14=O3⋯*Cg* = 95.3 (1)°; *Cg* is the centroid of the C8–C13 ring; symmetry code: (i) *x*, −*y* + 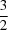, *z* + 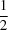], as shown in Fig. 3 ▸. There are also parallel slipped π–π inter­actions present (Fig. 4 ▸), involving inversion-related benzene rings (C15–C20) with a centroid–centroid distance of 3.8850 (14) Å [inter-planar distance = 3.3895 (10) Å; slippage = 1.899 Å]. The result of these inter­actions leads to the formation of sheets lying parallel to (100), as shown in Fig. 5 ▸.

## Synthesis and crystallization   

To a warm methano­lic solution (25 ml) of *N* ^4^-phenyl­semi­carbazide (0.302 g, 2 mmol), a methano­lic solution (25 ml) of 2-hy­droxy-4-meth­oxy­benzo­phenone (0.4566 g, 2 mmol) was added and the resulting solution was boiled under reflux for 2 h, after adding three drops of conc. HCl. On slow evaporation at room temperature, colourless crystals separated out. They were filtered off and washed with methanol and ether. Single crystals of the title compound suitable for X-ray analysis were obtained by slow evaporation of a solution in methanol (yield: 0.1735 g, 76%; m.p.: 498 K). FT–IR (KBr, cm^−1^) ν_max_: 3316 (*s*, OH), 3249 (*m*, NH), 3145 (*m*, NH), 1662 (*s*, C=O), 1631 (*m*, C=N), 1059 (*m*, N–N). ^1^H NMR (DMSO-*d*_6_, δ, p.p.m.): 12.94 (*s*, 1H, OH), 9.10 (*s*, 1H, NH), 9.03 (*s*, 1H, NH), 3.90 (*s*, 3H, OMe), 6.33–7.672 (*m*, 13H, Ar-H). ESI mass spectrum, *m*/*z*: 362.3 (*M*+1). Analysis calculated for C_21_H_19_N_3_O_3_: C, 69.79, H, 5.30, N, 11.63%. Found: C, 69.68, H, 5.72, N, 11.93%.

## Refinement   

Crystal data, data collection and structure refinement details are summarized in Table 2 ▸. The OH and NH H atoms were located in a difference Fourier map and refined with distances restraints of 0.88 (1) Å. The C-bound H atoms were placed in calculated positions and refined as riding atoms: C—H = 0.93–0.96 Å with *U*_iso_(H) = 1.5*U*_eq_(C) for methyl H atoms and 1.2*U*_eq_(C) for other H atoms.

## Supplementary Material

Crystal structure: contains datablock(s) I, Global. DOI: 10.1107/S2056989015005757/su5098sup1.cif

Structure factors: contains datablock(s) I. DOI: 10.1107/S2056989015005757/su5098Isup2.hkl

Supporting information file. DOI: 10.1107/S2056989015005757/su5098Isup3.cml

CCDC reference: 1055367

Additional supporting information:  crystallographic information; 3D view; checkCIF report

## Figures and Tables

**Table 1:** Hydrogen-bond geometry (, )

*D*H*A*	*D*H	H*A*	*D* *A*	*D*H*A*
O2H2*O*N1	0.89(1)	1.76(2)	2.563(2)	149(3)
N2H2*N*O3^i^	0.87(1)	2.13(1)	2.9301(19)	152(2)
N3H3*N*O3^i^	0.88(1)	2.09(1)	2.935(2)	161(2)
C12H12O2^ii^	0.93	2.44	3.252(3)	146

Symmetry codes: (i) 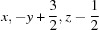; (ii) 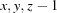.

**Table 2:** Experimental details

Crystal data	
Chemical formula	C_21_H_19_N_3_O_3_
*M*_r_	361.39
Crystal system, space group	Monoclinic, *P*2_1_/*c*
Temperature (K)	296
*a*, *b*, *c* ()	19.965(2), 9.9788(9), 9.3366(7)
()	90.340(5)
*V* (^3^)	1860.1(3)
*Z*	4
Radiation type	Mo *K*
(mm^1^)	0.09
Crystal size (mm)	0.28 0.24 0.21

Data collection	
Diffractometer	Bruker APEXII CCD
Absorption correction	Multi-scan (*SADABS*; Bruker, 2004 ▸)
*T*_min_, *T*_max_	0.955, 0.961
No. of measured, independent and observed [*I* > 2(*I*)] reflections	18641, 4268, 2092
*R*_int_	0.057
(sin /)_max_ (^1^)	0.650

Refinement	
*R*[*F* ^2^ > 2(*F* ^2^)], *wR*(*F* ^2^), *S*	0.049, 0.143, 1.00
No. of reflections	4240
No. of parameters	257
No. of restraints	3
H-atom treatment	H atoms treated by a mixture of independent and constrained refinement
_max_, _min_ (e ^3^)	0.17, 0.19

Computer programs: *APEX2*, *SAINT* and *XPREP* (Bruker, 2004 ▸), *SHELXS97* (Sheldrick, 2008 ▸), *SHELXL2014* (Sheldrick, 2015 ▸), *ORTEP-3 for Windows* (Farrugia, 2012 ▸), *DIAMOND* (Brandenburg, 2010 ▸), and *publCIF* (Westrip, 2010 ▸).

## supplementary crystallographic information

### Crystal data

**Table:**

C_21_H_19_N_3_O_3_	*F*(000) = 760.0
*M**_r_* = 361.39	*D*_x_ = 1.291 Mg m^−^^3^
Monoclinic, *P*2_1_/*c*	Mo *K*α radiation, λ = 0.71073 Å
Hall symbol: -P 2ybc	Cell parameters from 2338 reflections
*a* = 19.965 (2) Å	θ = 2.9–22.7°
*b* = 9.9788 (9) Å	µ = 0.09 mm^−^^1^
*c* = 9.3366 (7) Å	*T* = 296 K
β = 90.340 (5)°	Block, colourless
*V* = 1860.1 (3) Å^3^	0.28 × 0.24 × 0.21 mm
*Z* = 4	

### Data collection

**Table:**

Bruker APEXII CCD diffractometer	4268 independent reflections
Radiation source: fine-focus sealed tube	2092 reflections with *I* > 2σ(*I*)
Graphite monochromator	*R*_int_ = 0.057
ω and φ scan	θ_max_ = 27.5°, θ_min_ = 2.9°
Absorption correction: multi-scan (*SADABS*; Bruker, 2004)	*h* = −25→25
*T*_min_ = 0.955, *T*_max_ = 0.961	*k* = −12→12
18641 measured reflections	*l* = −10→12

### Refinement

**Table:**

Refinement on *F*^2^	Primary atom site location: structure-invariant direct methods
Least-squares matrix: full	Secondary atom site location: difference Fourier map
*R*[*F*^2^ > 2σ(*F*^2^)] = 0.049	Hydrogen site location: inferred from neighbouring sites
*wR*(*F*^2^) = 0.143	H atoms treated by a mixture of independent and constrained refinement
*S* = 1.00	*w* = 1/[σ^2^(*F*_o_^2^) + (0.0608*P*)^2^ + 0.0804*P*] where *P* = (*F*_o_^2^ + 2*F*_c_^2^)/3
4240 reflections	(Δ/σ)_max_ < 0.001
257 parameters	Δρ_max_ = 0.17 e Å^−^^3^
3 restraints	Δρ_min_ = −0.19 e Å^−^^3^

### Special details

**Table:**

Geometry. All esds (except the esd in the dihedral angle between two l.s. planes) are estimated using the full covariance matrix. The cell esds are taken into account individually in the estimation of esds in distances, angles and torsion angles; correlations between esds in cell parameters are only used when they are defined by crystal symmetry. An approximate (isotropic) treatment of cell esds is used for estimating esds involving l.s. planes.
Refinement. Refinement of F^2^ against ALL reflections. The weighted R-factor wR and goodness of fit S are based on F^2^, conventional R-factors R are based on F, with F set to zero for negative F^2^. The threshold expression of F^2^ > 2sigma(F^2^) is used only for calculating R-factors(gt) etc. and is not relevant to the choice of reflections for refinement. R-factors based on F^2^ are statistically about twice as large as those based on F, and R- factors based on ALL data will be even larger.

### Fractional atomic coordinates and isotropic or equivalent isotropic displacement parameters (Å^2^)

**Table:**

	*x*	*y*	*z*	*U*_iso_*/*U*_eq_	
N1	0.24470 (9)	0.62277 (18)	0.17814 (16)	0.0444 (4)	
N2	0.19511 (9)	0.68714 (18)	0.10330 (17)	0.0469 (5)	
O3	0.14953 (7)	0.76718 (14)	0.30843 (12)	0.0480 (4)	
O2	0.32524 (9)	0.62214 (17)	0.39293 (14)	0.0621 (5)	
C14	0.14865 (10)	0.7582 (2)	0.17789 (19)	0.0394 (5)	
N3	0.10153 (9)	0.81374 (18)	0.09207 (17)	0.0491 (5)	
O1	0.49774 (9)	0.31233 (18)	0.41036 (17)	0.0779 (6)	
C7	0.27941 (10)	0.5321 (2)	0.11323 (19)	0.0415 (5)	
C2	0.40967 (11)	0.4616 (2)	0.3955 (2)	0.0522 (6)	
H2	0.4235	0.4949	0.4838	0.063*	
C6	0.33388 (10)	0.4695 (2)	0.19314 (19)	0.0411 (5)	
C1	0.35550 (11)	0.5171 (2)	0.32703 (19)	0.0436 (5)	
C5	0.36938 (12)	0.3632 (2)	0.1362 (2)	0.0574 (6)	
H5	0.3562	0.3293	0.0476	0.069*	
C4	0.42306 (13)	0.3056 (2)	0.2043 (2)	0.0632 (7)	
H4	0.4452	0.2332	0.1635	0.076*	
C15	0.03853 (11)	0.8599 (2)	0.1368 (2)	0.0435 (5)	
C3	0.44366 (12)	0.3566 (2)	0.3338 (2)	0.0540 (6)	
C8	0.26680 (11)	0.4937 (2)	−0.03890 (19)	0.0434 (5)	
C20	−0.01559 (12)	0.8337 (2)	0.0487 (2)	0.0545 (6)	
H20	−0.0097	0.7861	−0.0359	0.065*	
C13	0.30627 (12)	0.5462 (2)	−0.1443 (2)	0.0596 (6)	
H13	0.3413	0.6035	−0.1204	0.072*	
C16	0.02980 (13)	0.9330 (2)	0.2607 (2)	0.0580 (6)	
H16	0.0663	0.9534	0.3192	0.070*	
C9	0.21564 (14)	0.4095 (3)	−0.0764 (2)	0.0700 (8)	
H9	0.1883	0.3734	−0.0059	0.084*	
C17	−0.03359 (15)	0.9754 (3)	0.2968 (3)	0.0700 (8)	
H17	−0.0398	1.0237	0.3809	0.084*	
C19	−0.07808 (14)	0.8781 (2)	0.0864 (3)	0.0682 (7)	
H19	−0.1144	0.8608	0.0265	0.082*	
C11	0.24422 (16)	0.4294 (3)	−0.3212 (3)	0.0796 (9)	
H11	0.2372	0.4064	−0.4166	0.096*	
C10	0.20429 (16)	0.3776 (3)	−0.2181 (3)	0.0865 (9)	
H10	0.1693	0.3205	−0.2429	0.104*	
C12	0.29420 (15)	0.5144 (3)	−0.2855 (2)	0.0732 (8)	
H12	0.3207	0.5516	−0.3568	0.088*	
C21	0.53543 (18)	0.2044 (3)	0.3538 (4)	0.1201 (14)	
H21A	0.5074	0.1265	0.3459	0.180*	
H21B	0.5726	0.1854	0.4164	0.180*	
H21C	0.5518	0.2283	0.2608	0.180*	
C18	−0.08757 (14)	0.9475 (3)	0.2108 (3)	0.0732 (8)	
H18	−0.1303	0.9754	0.2368	0.088*	
H2N	0.1922 (10)	0.6792 (19)	0.0104 (10)	0.050 (6)*	
H3N	0.1063 (11)	0.795 (2)	0.0004 (11)	0.058 (7)*	
H2O	0.2911 (10)	0.647 (3)	0.337 (3)	0.107 (11)*	

### Atomic displacement parameters (Å^2^)

**Table:**

	*U*^11^	*U*^22^	*U*^33^	*U*^12^	*U*^13^	*U*^23^
N1	0.0438 (11)	0.0557 (11)	0.0336 (9)	0.0068 (9)	−0.0022 (8)	0.0030 (8)
N2	0.0493 (12)	0.0655 (12)	0.0257 (9)	0.0148 (10)	−0.0033 (8)	0.0010 (8)
O3	0.0552 (10)	0.0646 (9)	0.0241 (7)	0.0057 (8)	−0.0020 (6)	−0.0009 (6)
O2	0.0721 (12)	0.0777 (11)	0.0363 (8)	0.0328 (10)	−0.0091 (8)	−0.0112 (8)
C14	0.0411 (13)	0.0493 (12)	0.0277 (10)	0.0010 (10)	−0.0007 (9)	0.0003 (9)
N3	0.0490 (12)	0.0733 (13)	0.0249 (9)	0.0157 (10)	−0.0021 (8)	0.0011 (8)
O1	0.0732 (13)	0.0986 (14)	0.0617 (10)	0.0413 (11)	−0.0122 (9)	−0.0005 (9)
C7	0.0430 (13)	0.0478 (12)	0.0337 (10)	−0.0016 (10)	0.0007 (9)	0.0023 (9)
C2	0.0576 (15)	0.0664 (15)	0.0326 (11)	0.0137 (13)	−0.0040 (10)	0.0012 (10)
C6	0.0449 (13)	0.0450 (12)	0.0335 (10)	0.0024 (10)	0.0007 (9)	0.0005 (9)
C1	0.0487 (14)	0.0509 (12)	0.0314 (10)	0.0094 (11)	0.0062 (9)	0.0031 (9)
C5	0.0663 (17)	0.0582 (14)	0.0476 (12)	0.0123 (13)	−0.0096 (12)	−0.0118 (11)
C4	0.0706 (18)	0.0619 (16)	0.0571 (14)	0.0247 (14)	−0.0020 (13)	−0.0077 (12)
C15	0.0489 (14)	0.0469 (12)	0.0347 (10)	0.0093 (11)	0.0040 (10)	0.0075 (9)
C3	0.0530 (15)	0.0617 (14)	0.0473 (13)	0.0181 (12)	−0.0004 (11)	0.0072 (11)
C8	0.0457 (13)	0.0489 (12)	0.0355 (11)	0.0019 (11)	−0.0007 (10)	−0.0007 (9)
C20	0.0558 (16)	0.0542 (14)	0.0534 (13)	0.0072 (12)	−0.0064 (12)	−0.0015 (10)
C13	0.0611 (17)	0.0745 (16)	0.0432 (12)	−0.0076 (14)	0.0038 (11)	−0.0028 (11)
C16	0.0703 (18)	0.0616 (15)	0.0421 (12)	0.0153 (13)	−0.0028 (11)	−0.0033 (11)
C9	0.078 (2)	0.0788 (18)	0.0527 (14)	−0.0260 (16)	−0.0001 (13)	−0.0057 (13)
C17	0.079 (2)	0.0709 (17)	0.0598 (15)	0.0280 (16)	0.0135 (15)	−0.0021 (13)
C19	0.0517 (17)	0.0654 (16)	0.0873 (19)	0.0040 (14)	−0.0091 (14)	0.0015 (15)
C11	0.094 (2)	0.103 (2)	0.0423 (14)	0.0101 (19)	−0.0110 (15)	−0.0219 (14)
C10	0.095 (2)	0.099 (2)	0.0659 (18)	−0.0282 (19)	−0.0122 (17)	−0.0209 (16)
C12	0.083 (2)	0.098 (2)	0.0383 (13)	0.0036 (18)	0.0053 (13)	0.0006 (13)
C21	0.116 (3)	0.138 (3)	0.106 (3)	0.086 (3)	−0.024 (2)	−0.019 (2)
C18	0.0589 (18)	0.0712 (18)	0.090 (2)	0.0184 (15)	0.0185 (16)	0.0137 (16)

### Geometric parameters (Å, º)

**Table:**

N1—C7	1.293 (2)	C15—C16	1.379 (3)
N1—N2	1.369 (2)	C8—C9	1.367 (3)
N2—C14	1.362 (3)	C8—C13	1.368 (3)
N2—H2N	0.873 (9)	C20—C19	1.372 (3)
O3—C14	1.222 (2)	C20—H20	0.9300
O2—C1	1.359 (2)	C13—C12	1.376 (3)
O2—H2O	0.890 (10)	C13—H13	0.9300
C14—N3	1.351 (2)	C16—C17	1.378 (3)
N3—C15	1.405 (3)	C16—H16	0.9300
N3—H3N	0.881 (9)	C9—C10	1.377 (3)
O1—C3	1.364 (3)	C9—H9	0.9300
O1—C21	1.417 (3)	C17—C18	1.369 (3)
C7—C6	1.456 (3)	C17—H17	0.9300
C7—C8	1.491 (3)	C19—C18	1.367 (3)
C2—C1	1.370 (3)	C19—H19	0.9300
C2—C3	1.376 (3)	C11—C12	1.350 (4)
C2—H2	0.9300	C11—C10	1.357 (4)
C6—C5	1.384 (3)	C11—H11	0.9300
C6—C1	1.403 (3)	C10—H10	0.9300
C5—C4	1.369 (3)	C12—H12	0.9300
C5—H5	0.9300	C21—H21A	0.9600
C4—C3	1.373 (3)	C21—H21B	0.9600
C4—H4	0.9300	C21—H21C	0.9600
C15—C20	1.379 (3)	C18—H18	0.9300
			
C7—N1—N2	118.52 (16)	C13—C8—C7	119.48 (19)
C14—N2—N1	118.42 (15)	C19—C20—C15	119.8 (2)
C14—N2—H2N	120.9 (13)	C19—C20—H20	120.1
N1—N2—H2N	120.6 (13)	C15—C20—H20	120.1
C1—O2—H2O	106.6 (19)	C8—C13—C12	120.1 (2)
O3—C14—N3	124.56 (19)	C8—C13—H13	119.9
O3—C14—N2	122.83 (18)	C12—C13—H13	119.9
N3—C14—N2	112.59 (16)	C17—C16—C15	119.2 (2)
C14—N3—C15	125.39 (17)	C17—C16—H16	120.4
C14—N3—H3N	114.4 (14)	C15—C16—H16	120.4
C15—N3—H3N	117.3 (14)	C8—C9—C10	120.4 (2)
C3—O1—C21	118.1 (2)	C8—C9—H9	119.8
N1—C7—C6	117.45 (17)	C10—C9—H9	119.8
N1—C7—C8	122.56 (18)	C18—C17—C16	121.0 (2)
C6—C7—C8	119.97 (18)	C18—C17—H17	119.5
C1—C2—C3	120.2 (2)	C16—C17—H17	119.5
C1—C2—H2	119.9	C18—C19—C20	120.8 (2)
C3—C2—H2	119.9	C18—C19—H19	119.6
C5—C6—C1	116.52 (19)	C20—C19—H19	119.6
C5—C6—C7	120.98 (18)	C12—C11—C10	120.0 (2)
C1—C6—C7	122.42 (18)	C12—C11—H11	120.0
O2—C1—C2	116.90 (18)	C10—C11—H11	120.0
O2—C1—C6	121.97 (18)	C11—C10—C9	120.0 (3)
C2—C1—C6	121.10 (19)	C11—C10—H10	120.0
C4—C5—C6	123.0 (2)	C9—C10—H10	120.0
C4—C5—H5	118.5	C11—C12—C13	120.5 (3)
C6—C5—H5	118.5	C11—C12—H12	119.8
C5—C4—C3	118.9 (2)	C13—C12—H12	119.8
C5—C4—H4	120.5	O1—C21—H21A	109.5
C3—C4—H4	120.5	O1—C21—H21B	109.5
C20—C15—C16	119.9 (2)	H21A—C21—H21B	109.5
C20—C15—N3	117.38 (19)	O1—C21—H21C	109.5
C16—C15—N3	122.7 (2)	H21A—C21—H21C	109.5
O1—C3—C4	125.0 (2)	H21B—C21—H21C	109.5
O1—C3—C2	114.7 (2)	C19—C18—C17	119.3 (3)
C4—C3—C2	120.3 (2)	C19—C18—H18	120.4
C9—C8—C13	118.92 (19)	C17—C18—H18	120.4
C9—C8—C7	121.58 (19)		
			
C7—N1—N2—C14	164.67 (18)	C5—C4—C3—O1	−177.4 (2)
N1—N2—C14—O3	0.2 (3)	C5—C4—C3—C2	1.7 (4)
N1—N2—C14—N3	−178.30 (17)	C1—C2—C3—O1	178.3 (2)
O3—C14—N3—C15	−17.1 (3)	C1—C2—C3—C4	−0.8 (4)
N2—C14—N3—C15	161.40 (19)	N1—C7—C8—C9	−79.3 (3)
N2—N1—C7—C6	177.23 (17)	C6—C7—C8—C9	102.6 (3)
N2—N1—C7—C8	−0.9 (3)	N1—C7—C8—C13	99.2 (3)
N1—C7—C6—C5	174.0 (2)	C6—C7—C8—C13	−78.9 (3)
C8—C7—C6—C5	−7.8 (3)	C16—C15—C20—C19	−1.4 (3)
N1—C7—C6—C1	−9.4 (3)	N3—C15—C20—C19	−179.1 (2)
C8—C7—C6—C1	168.75 (19)	C9—C8—C13—C12	0.2 (4)
C3—C2—C1—O2	−178.7 (2)	C7—C8—C13—C12	−178.4 (2)
C3—C2—C1—C6	−0.5 (3)	C20—C15—C16—C17	2.0 (3)
C5—C6—C1—O2	179.1 (2)	N3—C15—C16—C17	179.6 (2)
C7—C6—C1—O2	2.4 (3)	C13—C8—C9—C10	0.3 (4)
C5—C6—C1—C2	1.0 (3)	C7—C8—C9—C10	178.8 (2)
C7—C6—C1—C2	−175.72 (19)	C15—C16—C17—C18	−0.8 (4)
C1—C6—C5—C4	−0.1 (4)	C15—C20—C19—C18	−0.4 (4)
C7—C6—C5—C4	176.6 (2)	C12—C11—C10—C9	−1.4 (5)
C6—C5—C4—C3	−1.2 (4)	C8—C9—C10—C11	0.3 (4)
C14—N3—C15—C20	−139.5 (2)	C10—C11—C12—C13	1.8 (4)
C14—N3—C15—C16	42.9 (3)	C8—C13—C12—C11	−1.2 (4)
C21—O1—C3—C4	−0.9 (4)	C20—C19—C18—C17	1.6 (4)
C21—O1—C3—C2	−179.9 (2)	C16—C17—C18—C19	−0.9 (4)

### Hydrogen-bond geometry (Å, º)

**Table:**

*D*—H···*A*	*D*—H	H···*A*	*D*···*A*	*D*—H···*A*
O2—H2*O*···N1	0.89 (1)	1.76 (2)	2.563 (2)	149 (3)
N2—H2*N*···O3^i^	0.87 (1)	2.13 (1)	2.9301 (19)	152 (2)
N3—H3*N*···O3^i^	0.88 (1)	2.09 (1)	2.935 (2)	161 (2)
C12—H12···O2^ii^	0.93	2.44	3.252 (3)	146

Symmetry codes: (i) *x*, −*y*+3/2, *z*−1/2; (ii) *x*, *y*, *z*−1.
